# Hemothorax Complicating Hickman Line Placement in a Pediatric Patient: A Case Report of Early Recognition and Management

**DOI:** 10.7759/cureus.79344

**Published:** 2025-02-20

**Authors:** Feras Ayaz, Osamah Arafah, Khalid Aljonaieh, Norah Al Mallohi, Leen Alshibi

**Affiliations:** 1 Anesthesia, King Faisal Specialist Hospital and Research Centre, Riyadh, SAU

**Keywords:** bone marrow transplantation, central venous catheterization, hemothorax, hickman catheter, pediatric critical care

## Abstract

Central venous catheters (CVCs) are essential in managing pediatric patients requiring long-term venous access, particularly for bone marrow transplantation (BMT). Although generally safe, their insertion is not without risks. We report the case of a one-year-old boy with Severe Combined Immunodeficiency Disorder (SCID) who developed a life-threatening right-sided hemothorax during Hickman line placement. The patient presented with acute hemodynamic instability and pulseless electrical activity (PEA) following multiple failed cannulation attempts. Immediate resuscitation measures restored circulation, and imaging revealed a large hemothorax. A chest tube was inserted, draining 138 mL of blood, leading to clinical stabilization. This case highlights the importance of early complication recognition, imaging, and prompt intervention during central venous catheterization.

## Introduction

Central venous catheters (CVCs) are indispensable in modern medical care, particularly in managing critically ill patients and those undergoing specialized treatments such as bone marrow transplantation (BMT). Among these devices, Hickman catheters have emerged as a preferred choice for long-term venous access due to their subcutaneous tunneling and Dacron cuff, which reduce the risk of infection and improve catheter longevity [1,2]. These catheters facilitate the administration of chemotherapy, parenteral nutrition, antibiotics, and blood products, making them essential in pediatric oncology and immunocompromised populations [3].

Despite their benefits, the insertion of Hickman catheters carries inherent risks, including mechanical complications such as pneumothorax, hemothorax, and malposition, as well as delayed issues like catheter-related bloodstream infections (CRBSIs) and thrombosis [4]. These complications, although relatively infrequent, can lead to significant morbidity and mortality, particularly in vulnerable populations [5]. For this reason, the use of ultrasound guidance and strict adherence to aseptic protocols during insertion has been widely advocated to minimize such risks [6].

Here, we report the case of a one-year-old boy with Severe Combined Immunodeficiency Disorder (SCID) who experienced a life-threatening right-sided hemothorax during Hickman line placement. This case highlights the critical importance of vigilance and rapid intervention when managing complications associated with central venous catheterization.

## Case presentation

A one-year-old boy with SCID, weighing 8 kg, was admitted for an elective Hickman line insertion in preparation for a BMT. The patient was born full-term via normal vaginal delivery, with no prenatal or perinatal complications. His medical history was significant for regular intravenous immunoglobulin (IVIG) therapy and prophylactic antibiotics. A thorough preoperative evaluation revealed no significant anesthetic risks.

**Figure 1:**
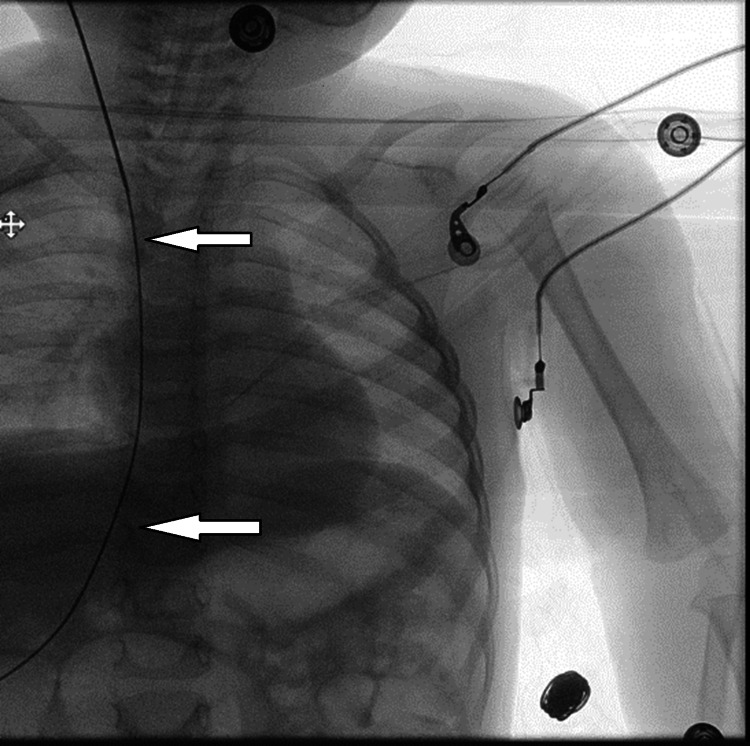
An anteroposterior chest X-ray, showing the guidewire of the catheter (white arrows) advancing into the inferior vena cava (IVC) via the right internal jugular vein, confirming its placement.

The procedure began with standard anesthetic induction using fentanyl, propofol, and ketamine via a 24G cannula placed in the right hand. A size 1.5 laryngeal mask airway (LMA) was inserted, and anesthesia was maintained with sevoflurane. Initial attempts at cannulating the left subclavian vein were unsuccessful, despite multiple attempts. The surgical team then opted for the right internal jugular vein, using ultrasound and fluoroscopic guidance to facilitate access. After several attempts, the guidewire was successfully inserted, and placement was confirmed via chest X-ray. However, further attempts to advance the catheter were unsuccessful (Figure 1).

Subtle signs of instability emerged during a subsequent attempt to reinsert the catheter. The patient displayed an increase in peak airway pressure, followed by the loss of the pulse oximeter signal. The pulse oximeter probe was replaced to rule out equipment malfunction, but the next cycle of non-invasive blood pressure (NIBP) monitoring yielded no readable values. Although the electrocardiogram (ECG) indicated a normal sinus rhythm, a carotid pulse check confirmed pulseless electrical activity (PEA).

An emergency code was called, and the patient was immediately intubated with a 3.5 cuffed endotracheal tube, secured at 12 cm at the lips. Bilateral equal air entry was confirmed via auscultation. Cardiopulmonary resuscitation (CPR) was initiated, and the patient received five doses of 50 mcg epinephrine. Fluid resuscitation included 80 mL of normal saline and 150 mL of 5% albumin. To facilitate further interventions, a left brachial arterial line was placed, and a left femoral CVC was inserted.

**Figure 2:**
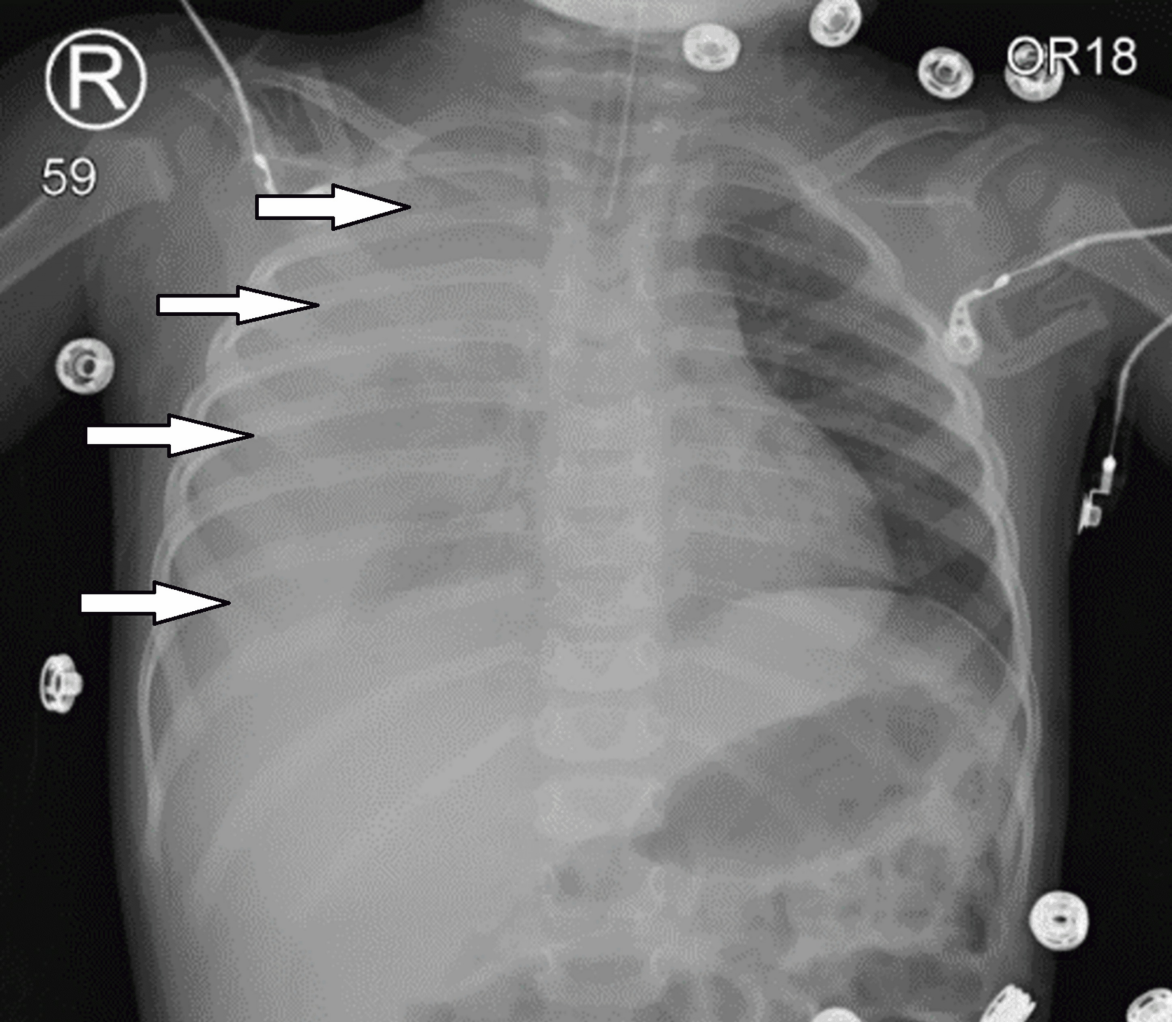
Post-ROSC anteroposterior chest X-ray showing right-sided hemothorax (white arrows). ROSC, return of spontaneous circulation

**Figure 3:**
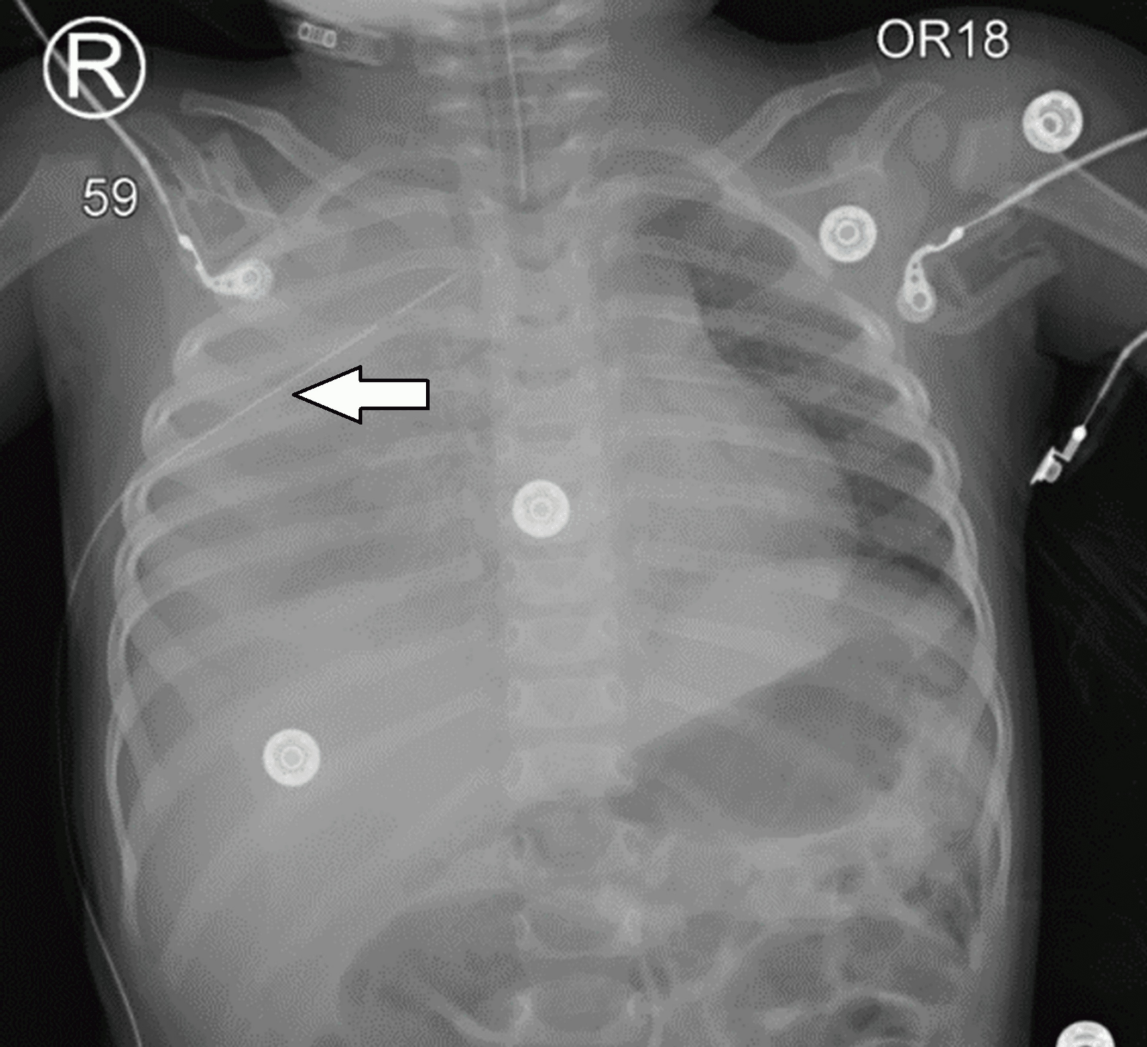
Anteroposterior chest X-ray showing right-sided hemothorax drainage with chest tube placement (white arrow).

After nine minutes of resuscitation, return of spontaneous circulation (ROSC) was achieved. Post-ROSC imaging revealed a large right-sided hemothorax (Figure 2). Arterial blood gas analysis demonstrated severe anemia, with a hemoglobin level of 56 g/L. A 12-French chest tube was inserted into the right thoracic cavity, immediately draining frank blood (Figure 3). To manage ongoing bleeding, the chest tube was intermittently clamped to maintain a tamponade effect.

The patient received an additional 20 mL of 5% albumin and 75 mL of packed red blood cells, resulting in hemodynamic stabilization. A total of 138 mL of fluid was drained intraoperatively. Following stabilization, the patient was transferred to the Pediatric Intensive Care Unit (PICU) for close monitoring and further care.

## Discussion

Hickman catheters are integral to the care of pediatric patients undergoing BMT. These devices provide reliable long-term venous access for administering chemotherapy, parenteral nutrition, medications, and blood products, while minimizing the need for repeated venipunctures [1]. However, their use is not without risks, as highlighted by this case.

The role of Hickman catheters in BMT cannot be overstated. They facilitate the administration of high-dose chemotherapy and other treatments directly into central circulation, ensuring effective and timely delivery. Additionally, they are indispensable for frequent blood sampling, transfusions, and total parenteral nutrition, particularly in patients with prolonged treatment regimens. The catheter's design, featuring subcutaneous tunneling and a Dacron cuff, reduces infection risks and ensures secure placement, making it suitable for long-term use [2,3].

In our case, a one-year-old boy with SCID required a Hickman catheter for BMT preparation. Despite a routine preoperative evaluation that indicated no significant anesthetic risks, complications arose during catheter insertion. Multiple failed attempts at vein cannulation led to a right-sided hemothorax, which was subsequently diagnosed via an anteroposterior chest X-ray. The hemothorax resulted in a mediastinal shift and severe instability, necessitating immediate resuscitation and placement of a chest tube. Following the intervention, the patient’s condition stabilized, and he was transferred to the PICU for further care.

This case emphasizes the delicate balance required when managing CVC insertions in pediatric patients, particularly those with underlying conditions like SCID. The need for precise technique and vigilance during insertion is paramount to preventing complications that may escalate into life-threatening emergencies.

Despite these advantages, complications can arise, categorized as immediate or delayed. Immediate complications, such as hemothorax, pneumothorax, or malposition, often occur during insertion. In this case, the hemothorax resulted from multiple unsuccessful attempts to cannulate the vein, eventually causing a breach in vascular integrity. This aligns with existing literature, which reports hemothorax as a rare but life-threatening complication during central venous catheterization [4,5].

Delayed complications, including CRBSIs, thrombosis, and mechanical issues, can emerge over time. Studies suggest that CRBSIs are the most common delayed complication, with rates varying depending on the duration of catheter use and adherence to maintenance protocols [6,7]. Strict aseptic techniques during insertion and routine care are critical for reducing these risks [8].

Imaging plays a pivotal role in the timely diagnosis of complications. In this case, the anteroposterior chest X-ray confirmed a large right-sided hemothorax, prompting immediate intervention. Such findings necessitate swift action to prevent further deterioration. Chest tube placement remains the cornerstone of hemothorax management, as evidenced by the patient’s stabilization after the drainage of frank blood through a 12-French chest tube [9].

Preventive strategies are essential to avoid such complications. Ultrasound guidance during catheter placement significantly reduces mechanical complications by providing real-time visualization of anatomical landmarks, increasing the success rate of first-attempt insertions. Additionally, proactive monitoring of catheter function and adherence to flushing protocols ensures long-term patency and reduces the risk of thrombosis and occlusion [10].

## Conclusions

Hickman catheters play a pivotal role in pediatric BMT, providing reliable access to essential treatments and reducing procedural burden. However, as this case demonstrates, their insertion is not without risks. Clinicians must maintain vigilance, employ preventive strategies such as ultrasound guidance, and prioritize prompt diagnosis and management of complications to optimize patient outcomes and ensure safety during these critical procedures.
